# Accumulation of TB-Active Compounds in Murine Organs Relevant to Infection by *Mycobacterium tuberculosis*

**DOI:** 10.3389/fphar.2020.00724

**Published:** 2020-05-19

**Authors:** Lloyd Tanner, Richard K. Haynes, Lubbe Wiesner

**Affiliations:** ^1^ Division of Clinical Pharmacology, Department of Medicine, University of Cape Town, Cape Town, South Africa; ^2^ Centre of Excellence for Pharmaceutical Sciences, Faculty of Health Sciences, North-West University, Potchefstroom, South Africa

**Keywords:** organ concentrations, tuberculosis, tuberculosis chemotherapy, decoquinate, phenoxazine, liquid chromatography-tandem mass spectrometry

## Abstract

Tuberculosis (TB), the leading cause of death due to an infectious agent, requires prolonged and costly drug treatments. With the rise in incidence of MDR and XDR TB, newer more efficacious treatments which are better able to permeate into the deeper recesses of the human lung where bacteria reside are urgently required. To this end, two new promising drug candidates, the decoquinate derivative RMB041 and the phenoxazine PhX1, were assessed for their abilities to permeate into specific murine organs. In particular, PhX1 permeation into the lungs and heart was notably efficient, as reflected in the high relative AUC values of 9669 ± 120.2 min/nmol/mg and 12450 ± 45.2 min/nmol/mg for lung and heart tissue, respectively. However, neither compound maintained a free concentration in the lung which exceeded the compound’s respective MIC_90_ values, indicating the importance of correcting for organ specific binding.

## Introduction

The global incidence of tuberculosis (TB), caused by *Mycobacterium tuberculosis* (*Mtb),* is slowly decreasing in response to the implementation of multi-drug treatment regimens, predominantly involving the four first-line drugs isoniazid (INH), rifampicin (RIF), ethambutol (EMB), and pyrazinamide (WHO, 2010; WHO, 2019). Despite this, in 2017, an estimated 10 million new cases, and 1.5 million deaths due to TB were reported (WHO, 2019).

In relation to drug efficacy, plasma concentrations of the drug are normally used as a positiveindicator. However, for TB, the situation is decidedly more complex. *Mtb* sequesters to different compartments including macrophages, eventually within necrotic granulomas where *Mtb* may be extracellular and metabolically quiescent, the inner surfaces of open cavities, where *Mtb* occurs within, and extracellular to, multiple cell types. In an extracellular environment, *Mtb* is protected from the host immune system and able to replicate freely. Bacilli reside in cells in superficial tissues of the lung and in other organs (Ramírez-Lapausa et al., 2015). Such a diversity of environments is associated with different physiological states of *Mtb*, including metabolically quiescent bacteria arising through the stringent response known as ‘non-replicating persisters.’ Thus, the development of chemotherapeutic regimens capable of adequately sterilizing bacteria in these diverse environments is a formidable challenge. TB drug discovery requires an examination of the target sites of pulmonary TB, plasma concentrations of TB drugs do not provide a reliable guide for estimates of drug concentration in TB lesions (Dartois, 2014; Horsburgh et al., 2015; Lenaerts et al., 2015). Intensive modeling of known drugs and experimental compounds revealed a correlation of CLogP with ability to penetrate the granuloma; that is, the more lipophilic compounds are better (Dartois, 2014; Lenaerts et al., 2015). On the other hand, although the polar TB drug ethambutol (EMB) elicits relatively poor activity *in vitro*, it is notably efficacious *in vivo*, and it efficiently partitions into caseous lesions, for reasons that are not fully understood (Zimmerman et al., 2017). Clearly then, studies that may provide data for selection of early lead compounds must focus in addition on carefully conducted permeation studies involving uptake into specific organs, effects on quiescent bacteria, and judicious *in vivo* assays (Voskuil et al., 2011; Sarathy et al., 2017; Zimmerman et al., 2017; Strydom et al., 2019).

Thus, it is argued that better predictions for efficacy of a compound against *Mtb in vivo* would be obtained by determining specific drug concentrations at the target organ (Müller et al., 2004; Tanner et al., 2018). Thus, studies assessing drug concentration in target organs are necessary, preferably at an early stage in the drug discovery process. This would also assist in determining the main metabolizing organs for an individual compound that should provide a better understanding of organ specific metabolite formation and toxicity. Ensuring that compound concentrations are monitored as free fractions in organ(s) in which the disease prevails will allow better prediction of efficacy, rather than relying on proxies of target site concentrations (Rizk et al., 2017).

It has already been shown that concentration of a particular drug in the lung for example may be higher than that in the plasma (Sonopo et al., 2015). Indeed, this trend is shown to obtain for the first-line TB drugs PZA, RIF, and INH whose concentrations in the lungs of uninfected primate species (baboons) were higher than in the plasma (Liu et al., 2010). Additional studies using a murine model that focus predominantly on lung tissue concentrations further support the disconnect between plasma and lung concentrations (Kjellsson et al., 2012; Lanoix et al., 2015; Prideaux et al., 2018).

In order to determine *in vivo* organ concentrations, two methods are available: these entail either invasive techniques of organ harvesting and microdialysis, and non-invasive instrumental techniques using positron emission tomography (PET), fluorescence molecular tomography (FMT), and magnetic resonance spectroscopy (MRS) (Langer and Müller, 2004)). The non-invasive methods allow for the semi-quantitative determination of organ drug concentrations and have been used extensively for evaluation of drug concentrations in sensitive tissues including the brain (Yang et al., 2009; Vasquez et al., 2011). However, there are limitations to these methods, including difficulties in determining unbound tissue concentrations, the radiometric labeling of compounds which may be difficult, and the limited sensitivity of the techniques (Lin, 2006). Invasive methods such as organ homogenization combined with liquid chromatography-tandem mass spectrometry (LC-MS/MS) have allowed for accurate determinations of both bound and unbound concentrations (Elmquist and Sawchuk, 2000; Langer and Müller, 2004). The methodology requires LC-MS/MS quantification of drug concentrations from standard curves prepared using blank organ homogenates (Tamvakopoulos et al., 2000; Mariappan et al., 2013; Takai et al., 2014).

Drug discovery and development is a multifactorial process that incorporates numerous parameters when assessing novel compounds. Recently, attempts have been made to bridge the gap between experimentally determined pharmacokinetic (PK) and pharmacodynamic (PD) parameters and use this knowledge to better understand how a drug might fare in human clinical trials. This can be achieved by looking more closely at unbound drug concentrations obtained at the target site. The importance of determining unbound concentration has been described by Morgan et al. as “one of the three pillars of survival” for progressing a compound down the drug discovery pipeline, along with sufficient binding to the target and target modulation (Morgan et al., 2012).

**Figure 1:**
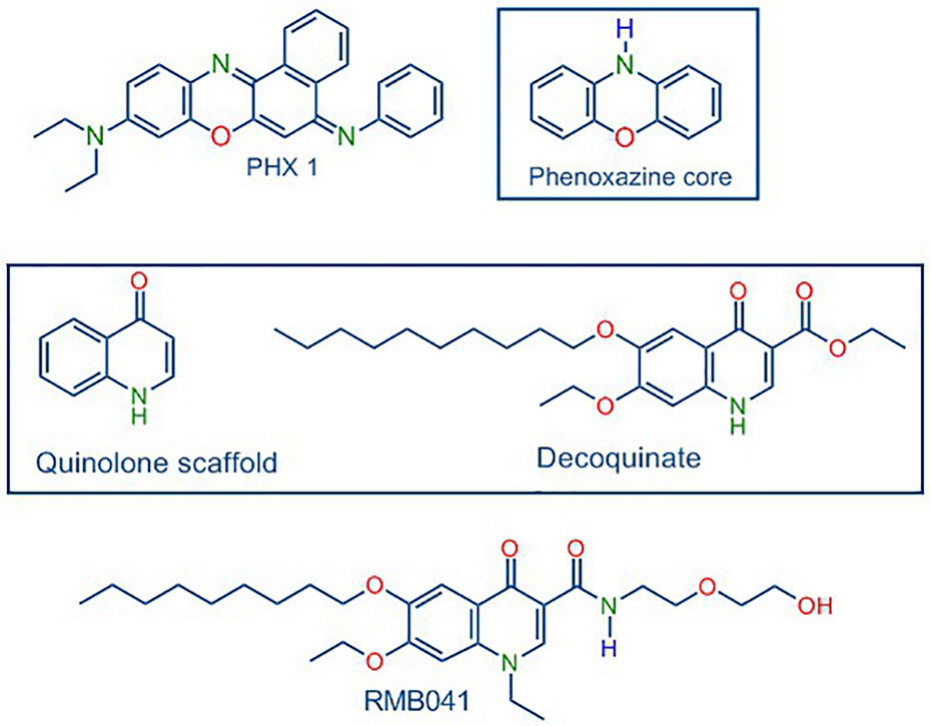
Structures of PhX1 incorporating the phenoxazine core, and RMB041 derived from decoquinate incorporating the quinolone core.

In turning to our own work, following in-depth *in vivo* murine studies, novel decoquinate derivative (RMB041) and a phenoxazine derivative (PhX1) (**Figure 1**) are now selected for murine organ analysis based on their promising *in vitro* ADME properties and their relatively long half-lives (62.5 ± 6.73 h and 3.71 ± 0.67 h) and large volumes of distribution (1.2 ± 0.03 L/kg and 11.1 ± 0.1 L/kg), respectively (Tanner et al., 2019a; Tanner et al., 2019b).

## Materials and Methods

### Ethics Statement

All animal studies were conducted with approval from the Animal Ethics Committee of the University of Cape Town (017/032). The experiments were conducted in accordance with the National Code for Animal Use in South Africa (National Research Council, 2011).

### Materials

The compounds RMB041 and PhX1 were synthesized and were shown by high-performance liquid chromatography (HPLC) analyses to be ≥96% pure (Shi et al., 2011; Beteck et al., 2018; Tanner et al., 2019a; Tanner et al., 2019b). Potassium dihydrogen phosphate and dipotassium hydrogen phosphate were purchased from Merck (Darmstadt, Germany). Analytical-grade acetonitrile (ACN) was purchased from Anatech (Johannesburg, South Africa). Analytical-grade dimethyl sulfoxide (DMSO), formic acid, and carbamazepine were purchased from Sigma-Aldrich (St. Louis, MO, USA). Water was purified *via* a Milli-Q purification system (Millipore, Bedford, MA, USA).

### *In Vivo* Organ Concentrations

#### Animals

Healthy male C57BL/6 mice, 12 to 16 weeks old, weighing approximately 30 g, were maintained at the Pharmacology satellite animal facility of the University of Cape Town. Mice were fed a standard laboratory diet and water was available *ad libitum*. Mice were housed in 27 cm × 21 cm × 18 cm cages, under controlled environmental conditions (26 ± 1°C with 12-h light/dark cycles). Mice were acclimatized to their experimental environment for 4 days before the experiment started.

#### Oral Drug Administration and Sample Collection

Clear suspensions of RMB041 and PhX1 were prepared in 100% hydroxypropyl methylcellulose. The compounds were administered *via* oral gavage at a dose of 10 mg/kg (n = 3 per time-point). The total volume administered per mouse was approximately 200 µl. At time points pre-determined by the PK study of these compounds, animals were anesthetized *via* intraperitoneal injection of ketamine/xylazine (75–100 mg/kg + 10 mg/kg) with depth of anesthesia monitored by the absence of the pedal withdrawal reflex. Blood samples, approximately 20 µl, were collected *via* tail bleeding 10 min before murine euthanasia at 1, 6, and 24 h post-drug administration. The organ collection was completed at set time points determined by the previous PK studies of the specific compounds (Tanner et al., 2019a; Tanner et al., 2019b). Following complete anesthesia, the mice (n = 3 per time-point) were then euthanized by exsanguination (cardiac puncture and the removal the majority of vascular blood), with both femoral arteries cut and approximately 20 ml of saline injected into the right aorta to rinse the circulatory system of blood, which continues until the organs experience a pale colour change which was followed by the surgical procedure. The area was shaved and washed before dissection along the mid-ventral line of the animal to expose the organs. The organs were then dissected out, weighed and flash frozen in liquid nitrogen, and stored at −80°C. To obtain blank matrix for calibration curve spiking, in order to determine concentrations of drug in the organs, the process was repeated in three mice that did not receive any compound.

#### Preparation of Calibration Standards and Quality Control Samples

Serial dilutions of each compound were spiked into blank murine organ homogenate to generate calibration standards (3.9–4000 ng/ml) and quality control (10–3200 ng/ml) samples (n = 3).

#### Sample Processing

Organ samples were homogenized using an Omni Bead Ruptor (Omni, Georgia, USA). The settings programmed for each organ are presented in **Table 1**. Organ samples were diluted 1:1 with PBS solution and homogenized according to the specific setting for each organ type. Blank homogenate samples were pooled in order to maximize organ volume for standard curve preparation.

**Table 1:** Homogenization parameters for organ experiments.

Parameter	Liver	Kidney	Lung	Spleen	Brain	Heart
Speed (m/s)	5	5.2	6	6	5	6
Cycle time (s)	30	30	30	30	30	30
Cycles	2	1	3	2	2	3
Dwell time (s)	10	10	10	10	10	10

After homogenization, organ samples (30 µl) were extracted using a liquid–liquid extraction method (LLE) using ethyl acetate (250 µl) and a 0.1-M Britton Robinson buffer (50 µl) at pH 4 for PhX1 and pH 10 for RMB 041, containing 1 µg/ml IS (carbamazepine) solution. Samples were vortexed (1 min) and centrifuged at 10,621*g* (5 min). Thereafter, 200 µl of the organic layer was removed and dried down under nitrogen. Samples were reconstituted in 150 µl of injection solvent (1:1 H_2_O: ACN) before being submitted for LC-MS/MS analysis.

#### LC-MS/MS Analysis

A reverse-phase HPLC column (Gemini NX, C18, 2.6 μm, 50 mm × 2.1 mm, Phenomenex) was used to separate the compounds and IS (mobile phase B, 0.1% FA in ACN; mobile phase A, 0.1% FA in analytical-grade water). Briefly, an Agilent 1200 Rapid Resolution HPLC system comprising a binary pump, degasser, and auto-sampler (Agilent, Little Falls, Wilmington, USA) coupled to an AB Sciex 4000 QTrap hybrid triple quadrupole linear ion-trap mass spectrometer (AB Sciex, Framingham, MA, USA) was used for sample analysis (**Table 2**). Gradient reversed-phase HPLC systems were used. For PhX1 an HPLC gradient using an elution profile consisting of mobile phase A (90%) for 0.5 min, decreased to 10% until 2 min, held at 10% mobile phase A for a further 1.6 min, then increased to 90% over 0.1 min, and finally held at 90% A for a further 2.3 min for a total run time of 6 min. For RMB041 an HPLC gradient using an elution profile consisting of mobile phase A (95%) for 0.5 min, decreased to 5% for a further 1.5 min, held at 5% mobile phase A for a further 1.6 min, then increased to 95% over 0.1 min, and finally held at 95% A for a further 2.3 min for a total run time of 6 min. The calibration curves generated were used to quantitatively determine the concentration of each analyte in the respective murine organ samples.

**Table 2:** MS/MS settings used for analysis of carbamazepine, RMB041, and PhX1 in mouse organ samples.

Parameter	RMB041	PhX1	Carbamazepine
Protonated precursor ion (m/z)	505.2	394.4	237.1
Product ion (m/z)	400.2	316.1	194.1
Ion spray voltage (V)	4,500	4,500	4,500
Nebulizer gas (AU)	40	40	40
Curtain gas (AU)	20	20	20
Turbo gas (AU)	20	20	20
Source temperature (°C)	400	400	400

AU, arbitrary unit.

#### Unbound Tissue Concentrations

Spiking solutions were diluted in phosphate buffer (final concentration = 1 μg/ml) and spiked into each respective organ tissue homogenate (total volume = 1 ml). Following vortexing (1 min), aliquots were transferred in duplicate to: (i) a final concentration plate that was immediately quenched with ACN containing internal standard (IS, carbamazepine (23.6 ng/ml)), (ii) a degradation control, which was placed in a water bath at 37°C for 4 h, and (iii) ultracentrifuge tubes, which were centrifuged for 4 h at 37°C and 30,000*g*. All reactions were stopped by the addition of ACN containing IS. The samples were subjected to liquid chromatography-tandem mass spectrometry (LC-MS/MS) analysis on an AB Sciex 4000 Q Trap hybrid triple quadrupole linear ion-trap MS (AB Sciex, Framingham, MA, USA) coupled to an Agilent 1200 HPLC (Agilent) with a reverse-phase Gemini-C18 analytical column (5 µm, 50 mm × 2 mm; Phenomenex) at 35°C. Mobile phases comprised 0.1% FA in water, and 0.1% FA in ACN. The flow rate was 600 µl/min with a run time of 6 min.

## Results

### Murine Organ Drug Analyses

#### LC-MS/MS Assay Performance

A quadratic regression equation, plotting peak area ratio against concentration was fitted to the calibration curves. The curves were weighted by 1/concentration (1/*x*). The accuracy (%NOM) for all calibration standards and QC samples was between 84.2 ± 4.1% and 117.7 ± 4.1% in this study, with precision (%CV) less than 15% for all samples. This indicated that the murine organ calibration curve performed well in the analysis of murine organ samples.

#### Murine Organ Concentrations of PhX1 and RMB 041

**Figure 2:**
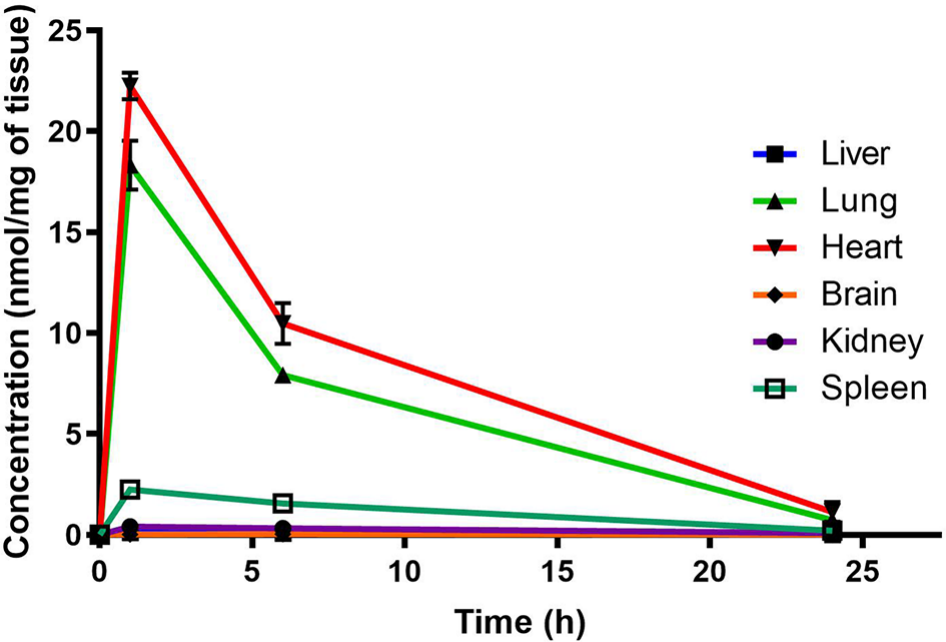
Total PhX1 concentrations in murine organs (oral dose, 10 mg/kg) (n = 3 per time-point; data presented as means ± SD).

Murine organ concentrations were determined as described above for PhX1 with the results presented in the figures [data presented as concentrations in nmol of compound/mg of tissue (**Figure 2**)]. Standard deviations were used to generate error bars for each time point (n = 3).

**Figure 3:**
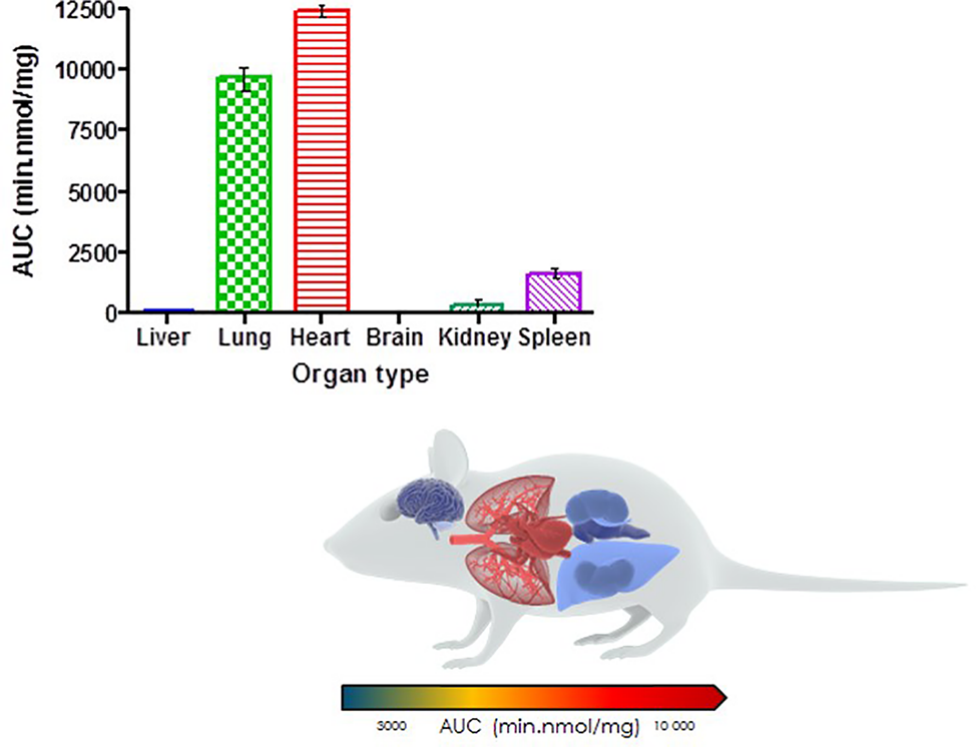
PhX1 exposure in each organ (AUC) (n = 3 per time-point; data presented as means ± SD).

Organ AUC was assessed by determining the area under the concentration time curve to provide values of exposure for each organ in the murine model over the 24-h testing period which is displayed in the bar graph and heat map of PhX1 in **Figure 3**. Organ concentrations obtained for PhX1 indicate drug accumulation within the lung (C_max_ 22.4 ± 2.35 nmol/mg) and heart (C_max_ 8.83 ± 3.16 nmol/mg) tissue.

**Figure 4:**
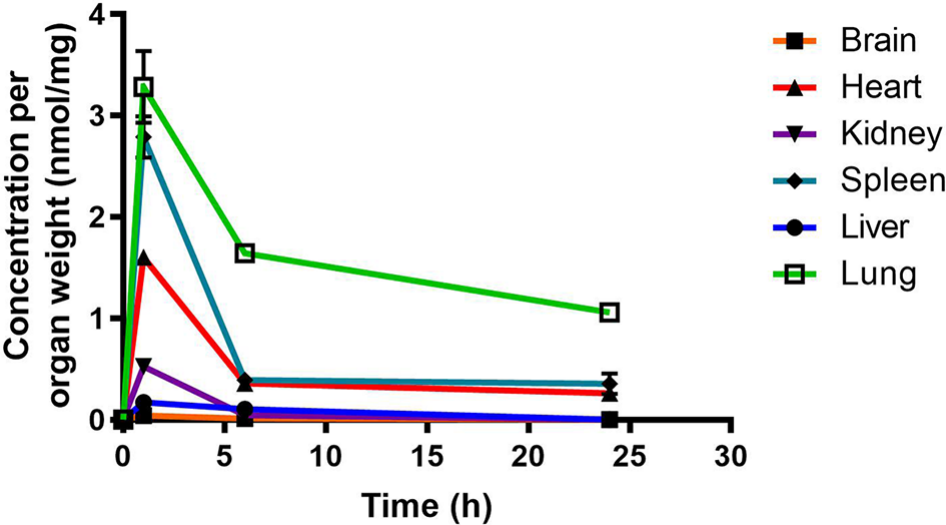
Total RMB041 concentrations in murine organs (10 mg/kg oral dose); (n = 3 per time-point; data presented as means ± SD).

RMB041 concentrations were assessed using the methodology described above for PhX1. This resulted in the concentration time curve produced below (**Figure 4**). Standard deviations were used to generate error bars for each time point (n = 3).

**Figure 5:**
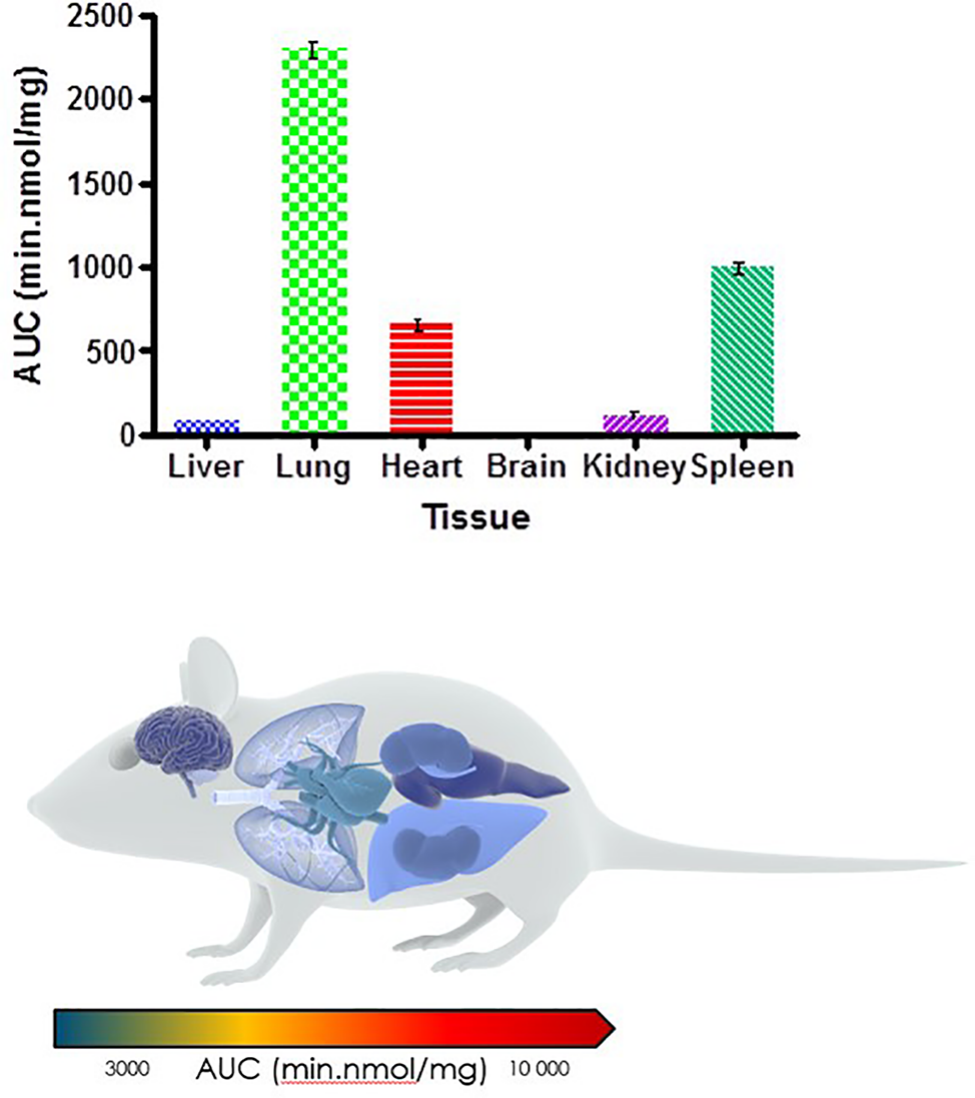
Total drug exposure (AUC) in organs after oral administration of RMB041 (n = 3 per time-point; data presented as means ± SD).

Murine exposures for RMB041 in each organ were assessed using the area under the concentration time curve in **Figure 4** and are presented in the bar graph and heat map below (**Figure 5**). Significantly lower concentrations of RMB041 were seen in all organs, with the lung tissue displaying the highest concentration (3.2 nmol/mg).

#### Free Drug Concentrations

It is essential to understand how much free drug is available to treat a specific microbe at the target site where the disease persists. In this study, drug concentrations were adjusted according to the protein binding experiments conducted for each tissue type (**Table 3**). Both PhX1 and RMB041 displayed unbound fractions between 0.18 and 0.01.

**Table 3:** PhX1 and RMB 041 bound ratio in specific tissue types.

Tissue type	Ratio bound PhX1	Ratio bound RMB041
Blood	0.88	0.82
Liver	0.99	0.91
Lung	0.97	0.93
Heart	0.98	0.97
Brain	0.98	0.98
Kidney	0.97	0.97
Spleen	0.94	0.92

**Figure 6:**
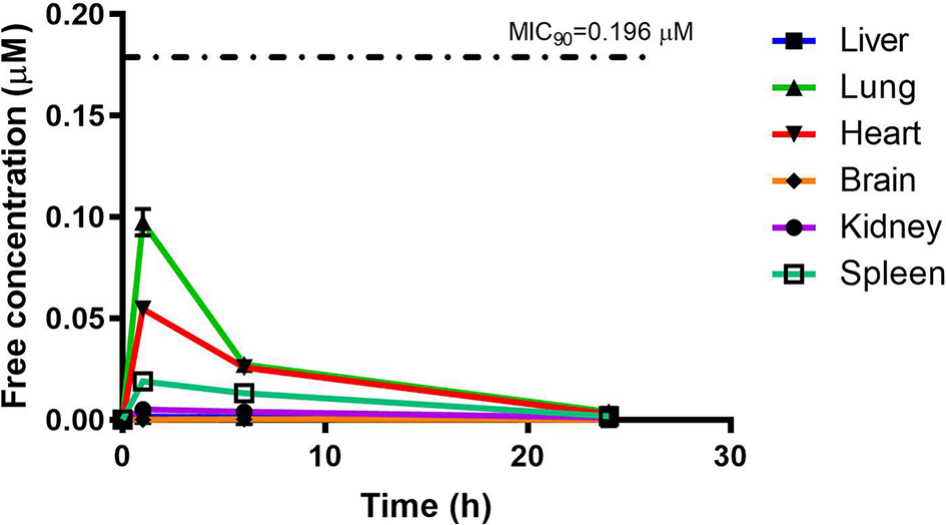
Free concentration of PhX1 (µM) following correction according to tissue binding.

PhX1 concentrations were assessed in each organ and corrected for using the fraction unbound value for each organ. This resulted in the concentration time curve produced below (**Figure 6**). Standard deviations were used to generate error bars for each time point (n = 3).

**Figure 7:**
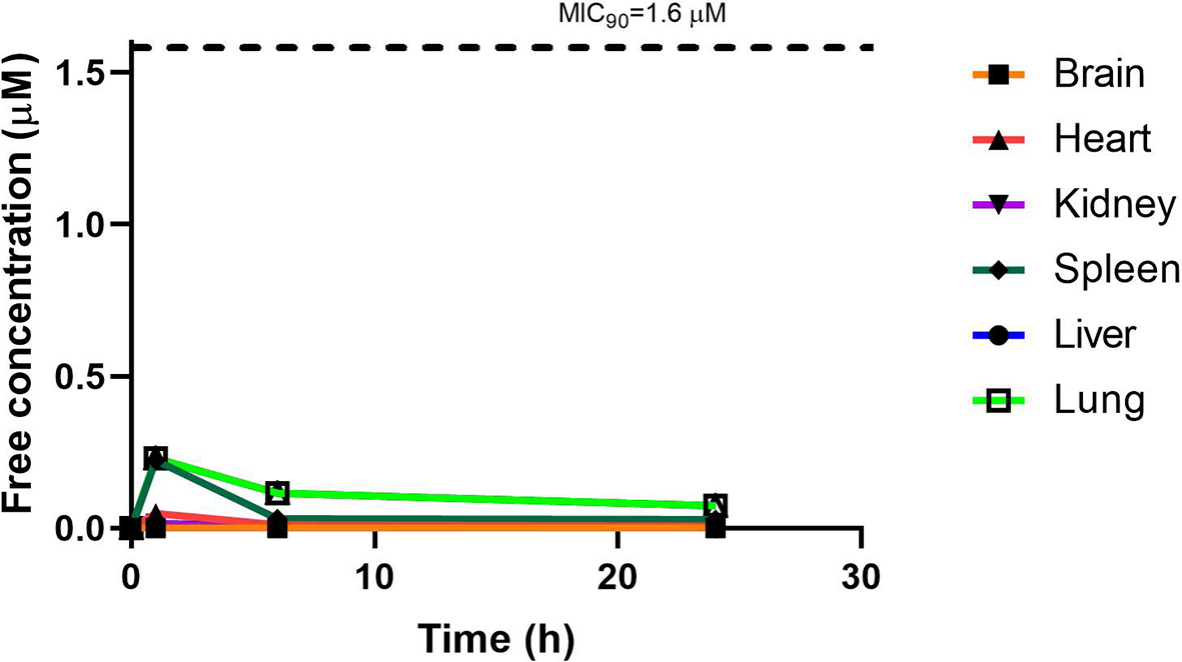
Free concentration of RMB041 (µM) following correction according to tissue binding.

RMB041 concentrations were assessed in each organ as indicated above and corrected for using the fraction unbound value for each organ, resulting in the concentration time curve produced below (**Figure 7**). Standard deviations were used to generate error bars for each time point (n = 3).

## Discussion

The decoquinate derivative, RMB041, and the phenoxazine derivative, PhX1, displayed significantly different concentrations in each murine organ. Concentrations of PhX1 were significantly different at the 1- and 6-h time-points with concentrations in the heart and lungs, decreasing to similar concentrations seen in other organs at the 24-h time point. Compound exposure in the lungs and heart was also higher with AUC values of 9669 ± 120.2 min/nmol/mg and 12450 ± 45.2 min/nmol/mg for lung and heart tissue, respectively. Significantly lower concentrations were reported in the murine kidney, spleen, brain, and liver for PhX1. These results are consistent with findings from murine experiments using the structurally-related riminophenazine antibiotic clofazimine (CLZ; Baik et al., 2013). Long-term drug exposure over a period of 8 weeks resulted in accumulation of CLZ into the spleen, lungs, and other organs, leading to the formation of crystal-like drug inclusions (CLDIs). This study focused on a 24-h period of standardized dosing to investigate detectable drug accumulation, while the study by Baik et al. included intensive dosing over 3 to 8 weeks and a follow-up washout period (Baik et al., 2013). Despite these differences similar trends were seen in accumulation between PhX1 and CLZ in the heart and lung tissues. Moreover, the brain and kidneys showed no significant drug accumulation at any stage (**Figures 2** and **3**). Baik et al. postulated that accumulation of drug in the spleen following cessation of drug administration may be due to immunomodulated accumulation of CLZ in macrophages (Baik and Rosania, 2012), a characteristic also observed after PhX1 administration in this study. Both compounds are planar aromatic molecules and are lipophilic and weakly basic (Wan et al., 2003). As such, these drugs, with their lysosomal trapping capacity, tend to accumulate in acidic organelle compartments *via* pH-dependent ion trapping mechanisms, potentially explaining accumulation in specific organ compartments (De Duve et al., 1974).

RMB041 accumulated to significantly lower levels than PhX1 in all organs, with the greatest concentrations observed in the lungs, spleen, and heart. Relatively low levels of RMB041 were seen in the murine liver, kidney, and brain. It must be noted that RMB041, although achieving lower maximum concentrations in all organs, displayed similar concentrations to PhX1 after 24 h, particularly in the lungs. Compounds that maintain concentrations in tissues for a prolonged period have a greater chance of clearing an infectious agent, such as *Mtb.* This is supported by the long half-life of RMB041 both *in vitro* and *in vivo*, respectively (Tanner et al., 2019b). Alternative explanations for the extended half-life of RMB041 accompanied by minimal drug accumulation in the murine organs, includes the accumulation of compound into adipose tissues (Hartmann et al., 2016) and organs, which were not assessed in this study. These results showed that the greatest exposure of RMB041 in murine organs over 24 h was observed in the lungs, spleen, and heart. However, these exposures were significantly lower than those obtained for PhX1.

Data from comparative studies for compounds related to those in this study are scarce, as only plasma concentrations of DQ, a quinolone derivative (**Figure 1**), have been assessed (Li et al., 2017). A study involving the quinolone antibiotics LVX, ciprofloxacin (CIP), and ampicillin, administered at a significantly higher dose (120 mg/kg) in a *Streptococcus pneumoniae*-infected mouse model, showed large maximal lung concentrations of 5.95, 1.10, and 1.71 µg/g, respectively (Ishida et al., 1999). This is supported by evidence of the uptake of fluoroquinolones into human-derived macrophage cells (Carryn et al., 2002; Michot et al., 2005; Vallet et al., 2018). The fluoroquinolones, however, differ significantly in their ability to permeate into cells and tissues, and even small changes in the structure of the compound could lead to differences in accumulation, as observed for RMB041.

Administration of higher or multiple doses of either of these compounds may lead to increased concentrations in the lungs and, with very little brain or liver exposure, the risk of toxicity is low. A concern arising from this study was the relatively high exposure of PhX1 in cardiac tissue. As it is unknown whether these compounds may cause human ether à-go-go-related gene (hERG) inhibition, which could interfere with electrical conductivity in the heart and result in arrhythmic conditions such as drug-induced QT prolongation. Confirmation of compound-related hERG toxicity is advisable before increasing the dose (Raschi et al., 2009).

Although PhX1 seemed to accumulate significantly within the lungs and heart, the total concentration of both compounds was significantly below the MIC_90_ level of 0.196 µM for PhX1 and 1.61 µM for RMB041 (**Figures 6** and **7**). It is important to develop an understanding of how the compound may behave at the target site of pulmonary TB (Tanner et al., 2018). The estimated organ free fraction concentration values for PhX1 and RMB041 were below the MIC_90_ values for both compounds (**Figures 6** and **7**). This finding is particularly important given the concentration-dependent nature of PhX1 killing (data not shown), with PhX1 present in the lungs at a concentration lower than the estimated free lung MIC_90_ value possibly indicating diminished efficacy within this lung environment (**Figure 6**). The time-dependent nature of RMB041’s mode of killing, with the free organ concentrations never above the MIC_90_ value points to a decreased level of killing in the lung environment (**Figure 7**). The MIC_90_ value for each compound assessed in this study may also be very different in the clinical *in vivo* situation.

The practice of comparing tissue concentrations determined in healthy animals to MIC values determined in different experiments has been criticized by Mouton and colleagues, who have stated that such data sets are not comparable, and that disease state could significantly alter the values obtained in these experiments (Mouton et al., 2008). In order to better utilize these data, the compounds assessed could be compared to other drugs known to accumulate significantly in healthy animal tissues. For example, bedaquiline (BDQ), a clinically successful drug, administered at 25 mg/kg accumulates to significant levels within healthy murine lung tissue (C_max_, 24 µM) but is also highly protein bound, leaving 0.18 µM free drug available, a value that is slightly higher than that for PhX1 and significantly higher than that for RMB041 (Irwin et al., 2016). Translating these data to clinical TB treatment becomes difficult, as changes in disease state within the same patient influence the protein binding over time (Grainger-Rousseau et al., 1989). This makes the interpretation of fraction unbound concentrations significantly more challenging in isolated use (Smith et al., 2010). However, comparing the MIC values to the free fraction offers some insight into the *in vivo* potency of the compound (Deitchman et al., 2018).

Testing these compounds in an *Mtb*-infected murine model would provide the chance to measure MIC values and drug concentrations in infected mice. This would provide a clearer understanding of whether *in vivo* efficacy and accumulation are influenced by the introduction of *Mtb* infection. These experiments would also allow for the continuous assessment of plasma protein binding over time in an *in vivo* situation. Administration of multiple or higher doses could also be assessed over a longer period to evaluate drug accumulation in a more clinically relevant situation, as few drugs are ever administered once-off and measured for 24 h in clinical practice.

## Conclusion

The murine organ analysis provided an insightful glimpse into the exposure of each murine organ to PhX1 and RMB041, the two compounds that yielded the most promising results in murine blood PK experiments. PhX1 showed significant accumulation in the heart and lung tissues, whilst RMB041 accumulated in these tissues to a significantly lesser extent, although neither compound produced free fractions greater than its respective MIC_90_ values. Progression into an infected murine model coupled with multiple-dosing would allow for a more clinically relevant estimate of drug concentrations in each organ.

## Data Availability Statement

All datasets generated for this study are included in the article/supplementary material.

## Ethics Statement

The animal study was reviewed and approved by Animal Ethics Committee of the University of Cape Town (017/032).

## Author Contributions

LT, RH, and LW were responsible for the conceptualization and design of the study. LT performed the experiments and analyzed and interpreted the data. LT drafted the manuscript and developed the figures and tables. All authors were involved in revising and approved the final version of the manuscript.

## Funding

We also thank SAMRC with funds from National Treasury under its Economic Competitiveness and Support Package for the North-West University (NWU) Flagship Project MAL-TB Redox (awarded to RH), and the NRF for funding provided to LT.

## Conflict of Interest

The authors declare that the research was conducted in the absence of any commercial or financial relationships that could be construed as a potential conflict of interest.
